# Microrefugia and Shifts of *Hippophae tibetana* (Elaeagnaceae) on the North Side of Mt. Qomolangma (Mt. Everest) during the Last 25000 Years

**DOI:** 10.1371/journal.pone.0097601

**Published:** 2014-05-19

**Authors:** Lu Xu, Hao Wang, Qiong La, Fan Lu, Kun Sun, Yang Fang, Mei Yang, Yang Zhong, Qianhong Wu, Jiakuan Chen, H. John B. Birks, Wenju Zhang

**Affiliations:** 1 Institute of Biodiversity Science, School of Life Sciences, Fudan University, Shanghai, China; 2 College of Life Sciences, Northwest Normal University, Lanzhou, China; 3 Department of Biology, Tibet University, Lhasa, China; 4 Department of Biology, University of Bergen, Bergen, Norway; 5 Environmental Change Research Centre, University College London, London, United Kingdom; 6 School of Geography and the Environment, University of Oxford, Oxford, United Kingdom; Agharkar Research Institute, India

## Abstract

Microrefugia at high altitudes or high latitudes are thought to play an important role in the post-glacial colonization of species. However, how populations in such microrefugia have responded to climate changes in alternating cold glacial and warm interglacial stages remain unclear. Here we present evidence to indicate the Rongbuk Valley of the Mt. Qomolangma (Mt. Everest) area, the highest region on earth, had microrefugia for *Hippophae tibetana* and discuss how this low shrub was adapted to the extreme climate fluctuations of the last 25,000 years by shifts. By integrating geological, glaciological, meteorological, and genetic information, we found that the Rongbuk Valley was not only a glacial microrefugium but also an interglacial microrefugium for *H. tibetana*: the former was located on the riverbank below 4800 m above sea level (asl) or lower area and the latter at ∼5000 m asl. Our results show that after the Last Glacial Maximum (LGM), *H. tibetana* in the valley has undergone upward and downward migrations around ∼5000 m driven by climate fluctuations and the population in the glacial microrefugium has suffered extinction or extreme contraction. Moreover, with the rise of temperature in the last four decades, the upper limit of *H. tibetana* has shifted at least 30 m upward. Combining population history and recent range shift of this species is important in predicting the fate of this endemic species to future climate changes.

## Introduction

The alternation between glacial and interglacial stages during the Quaternary has greatly affected species distributions [1], [2], [3]. Many temperate species retreated to refugia at lower latitudes or altitudes during glacials and expanded to higher latitudes or altitudes during interglacials [1], [4], [5], [6]. Apart from these broad-scale refugia, more and more scattered small refugia have been found at high latitudes or in alpine areas in recent decades, with local favourable environmental features outside the species’ main distribution area, so-called microrefugia (or cryptic refugia) [3], [4], [5], [7], [8], [9], [10], [11], [12]. Understanding microrefugia is of critical importance in interpreting species genetic diversity and evolutionary processes such as adaptation, speciation, and extinction [1], [2], [3], [9], [13], [14], [15], [16]. For example, the incongruence between estimated post-glacial migration rates and tree dispersal capacity (‘Reid’s Paradox’) can be explained, in part, by the widespread existence of microrefugia [5], [14], [15], [17], [18]. The concept of microrefugia is now well recognized through empirical studies which have mainly focused on identifying microrefugia during the Last Glacial Maximum (LGM) by palaeoecological or phylogeographical methods [4], [5], [10], [17]. However, as Rull (2009) points out, the concept of microrefugia lacks appropriate biogeographical and ecological characterization [9]. Although the importance of landscape physiography and microclimate for plant microrefugia to occur has been highlighted [15], and some potential microrefugia may be deduced according to topoclimate, climate stability, and isolation from the matrix [19], little is known about actual microrefugial situations, their time span, and their exact elevation or location, as well as how species (especially plants) have managed to track a favourable microclimate for their survival [8], [9], [15], [16], [20].

At present, most studies on microrefugia have paid close attention to glacier refugia, especially to the LGM microrefugia, in which some genotypes have been supposed to survive this cold period [4], [5], [7], [8], [9], [10], [14], [17], [21], [22], [23]. But as Hampe et al. (2013) pointed out that, with wide-ranging implications, glacial refugia no longer exist and can hence only be inferred by indirect means [16]. Populations in microrefugia, defined as climate relicts by Hampe et al. (2011) [24], should be the key to understanding the effects of microrefugia. An important fact often has been ignored by many researchers: although populations in the LGM microrefugia were able to persist through this cold period, these populations may have been dramatically changed in interglacial stages because of warm climate (e.g. American pika) [25]. Thus,when we infer glacial refugia by indirect means, especially by genetic information, the dynamics of these climate relics after the LGM are needed to survey at the smaller scale. A meta-population model has been considered to characterize these climate relicts [26]; however, few “climate relict” has been studied carefully in detail.

On the other hand, investigating species’ responses to past climate changes is also important in understanding how species might respond to recent and future climate changes [4], [5], [16], [18], [20], [27]. Global temperature has increased 0.6°C in the past three decades and 0.8°C in the past century [28], which has led to both latitudinal and altitudinal shifts in species ranges [29], [30], [31], [32], [33], [34] and even caused some species to be possibly on the brink of extinction [35], [36]. However, present studies on the responses of species to past climate changes and recent warming are very disconnected. The former often focuses on tracking species population history through phylogeographical or paleoecological methods whereas the latter usually involves comparison of well-documented historical records with present distributional data [29], [36], [37]. According to oxygen isotope (δ^18^O) data from ice cores and sea sediments, past climate oscillations are often more pronounced than recent warming [38], [39], [40], [41], but almost all the species existing today have survived these past oscillations [1], [5]. Thus, we could better predict a species response in the future by combining the present and past responses of the species to climate changes over a range of time scales.

In the present study, we investigate *Hippophae tibetana* Schlecht. (Elaeagnaceae) as a means of characterizing the biogeographical and ecological features of a particular microrefugium and the responses of this species to climate changes since the LGM and to recent warming. *H. tibetana* is a small dioecious shrub propagated by seeds or by horizontal roots [42]. It is endemic to the Qinghai-Tibet Plateau (QTP) and ranges from the west Himalaya to the east-north QTP [43]. In the eastern plateau, *H.tibetana* occurs in the lowlands and in alpine meadows at an altitude below 4000 m, but in the central plateau and the Himalayas, it has a fragmented distribution along several valleys. It is one of the shrubs to occur at the highest altitudes [44], growing up to ∼5200 m asl. Our previous study [43] investigated the phylogeography of *H. tibetana* and found that three main lineages (A, B, and C) of the present populations of this species occupy the middle, the western, and the eastern parts of its geographical range, respectively. Based on the distribution of a large number of private haplotypes, we concluded that *H. tibetana* had multiple LGM microrefugia on the Plateau and inferred that the Rongbuk Valley, north of Mt. Qomolangma (Mt. Everest) is a possible microrefugium for *H. tibetana* even though it is the highest region within the geographical range of *H. tibetana*. However, in this valley, where and how *H. tibetana* has survived since the LGM remains unclear. Also, we chose this area for our current study because the area is highly sensitive to global climate change, and the geological and meteorological characteristics of the valley have already been studied well [45], [46], [47], [48]. This area provides an excellent opportunity to consider the favourable local environment required for microrefugia to occur and to study the responses of *H. tibetana* to climate change since the LGM [4], [8] by integrating the available geological, meteorological, and genetic information. In the present study, we hope to clarify two questions: 1) Do microrefugia of the LGM for *H. tibetana* really exist in the Rongbuk valley, one of the highest areas on earth? 2) On the local scale, how has the population of *H. tibetana* in the microrefugia responded to climate changes since the LGM?

## Materials and Methods

### Ethics Statement

In this study, all field works were carried out in the Rongbuk Valley of Mountain Qomolangma National Nature Preserve (QNNP) and were permitted by QNNP. There is no endangered or protected species involved in this study. The plant species studied in this work, *Hippophae tibetana* (Elaeagnaceae), has a large distribution on the QTP and has not been listed in any protection lists. No animals were used in this study. Coordinate data of sample locations of this study were shown in Table 1.

**Table 1:** List of populations (POP) and patches (PAT) analysed in the present study with their sampling localities, number of specimens, coordinates, genetic diversity parameters, and chlorotype composition of each population.

Population No.	*N*	Altitude (m)	Latitude (N)	Longitude (E)	Nuclear microsatellites				Chloroplast Haplotypes		
					*F*_IS_	A	*H*_O_	*H*_E_	Haplotypes (Frequencies, %)	*D*	π
POP 1	39	4200	28°24′23″	86°59′14″	−0.061	2.10	0.419	0.390	R1(5), R2(85), R3(10)	0.2780	0.00053
POP 2	23	4400	28°18′49″	86°55′53″	−0.041	2.18	0.430	0.403	R1(44), R2(39), R3(17)	0.6561	0.00148
POP 3	51	4465	28°18′46″	86°53′39″	0.084	2.15	0.328	0.354	R1(39), R2(49), R3(12)	0.6039	0.00127
POP 4	25	4690	28°16′38″	86°48′26″	−0.048	2.28	0.410	0.384	R1(52), R2(36), R3(12)	0.6100	0.00135
POP 5	31	4805	28°14′38″	86°49′05″	0.031	2.25	0.422	0.429	R1(36), R2(61), R4(3)	0.5140	0.00098
POP 6	28	4946	28°12′25″	86°49′19″	−0.850	1.69	0.564	0.304	R1(100)	0.0000	0.00000
POP 7	33	5000	28°10′03″	86°50′23″	0.011	2.15	0.405	0.403*	R1(30), R2(3), R3(18), R4(12), R5(6), R6(31)	0.7879	0.00343
POP 8	35	5035	28°09′42″	86°50′38″	−0.051	2.19	0.429	0.402	R1(57), R2(5), R3(26), R4(11)	0.6084	0.00201
PAT 1	25	>5035	28°09′08″	86°50′52″	−0.778	1.20	0.176	0.099*	R3(100)	0.0000	0.00000
PAT 2	22	5047	28°09′34″	86°50′42″	−0.707	1.40	0.305	0.177*	R3(100)	0.0000	0.00000
PAT 3	15	5046	28°09′35″	86°50′42″	−0.774	1.49	0.373	0.209*	R2(100)	0.0000	0.00000
PAT 4	24	5047	28°09′37″	86°50′42″	−0.378	1.80	0.426	0.305*	R1(100)	0.0000	0.00000
PAT 5	32	5066	28°09′24″	86°50′49″	−1.000	1.20	0.200	0.100*	R2(100)	0.0000	0.00000
PAT 6	16	>5035	28°09′21″	86°50′56″	−0.408	1.78	0.390	0.267*	R2(100)	0.0000	0.00000
PAT 7	15	5058	28°09′32″	86°50′52″	−0.647	1.60	0.373	0.224*	R1(100)	0.0000	0.00000

The exact elevations of PAT 1 and PAT 6 are not given because the GPS data of the two spots are not very accurate. *N*, number of individuals analysed; Nuclear microsatellites data, including *F*_IS_, fixation index; A, allelic richness; *H*_O_, observed heterozygosity; *H*_E_, expected heterozygosity;

*significant Hardy–Weinberg disequilibrium. cpDNA haplotypes and their frequencies, as well as estimates of gene diversity (*D*) and nucleotide diversity averaged across loci (*π*) of the populations and patches studied.

### Study Area

The Rongbuk Valley is located on the northern slopes of Mt. Qomolangma (27.98°N, 86.92°E; elevation 8844 m, the highest mountain on Earth), which lies toward the eastern end of the Himalaya. Three large glaciers occur at the upper end of the valley: West Rongbuk Glacier, Rongbuk Glacier, and East Rongbuk Glacier. These glaciers flow for 13∼18 km down to the proglacial plain at 5200 m asl near Everest Base Camp used by many mountaineering expeditions [49], [50]. Glacial meltwater runs through the Rongbuk Valley, which is 0.4−1 km wide, ∼91 km long, and ∼1500 m fall at altitude (5200 m to 3700 m), and empties into the Pengqu River at 3700 m asl.

**Figure 1:**
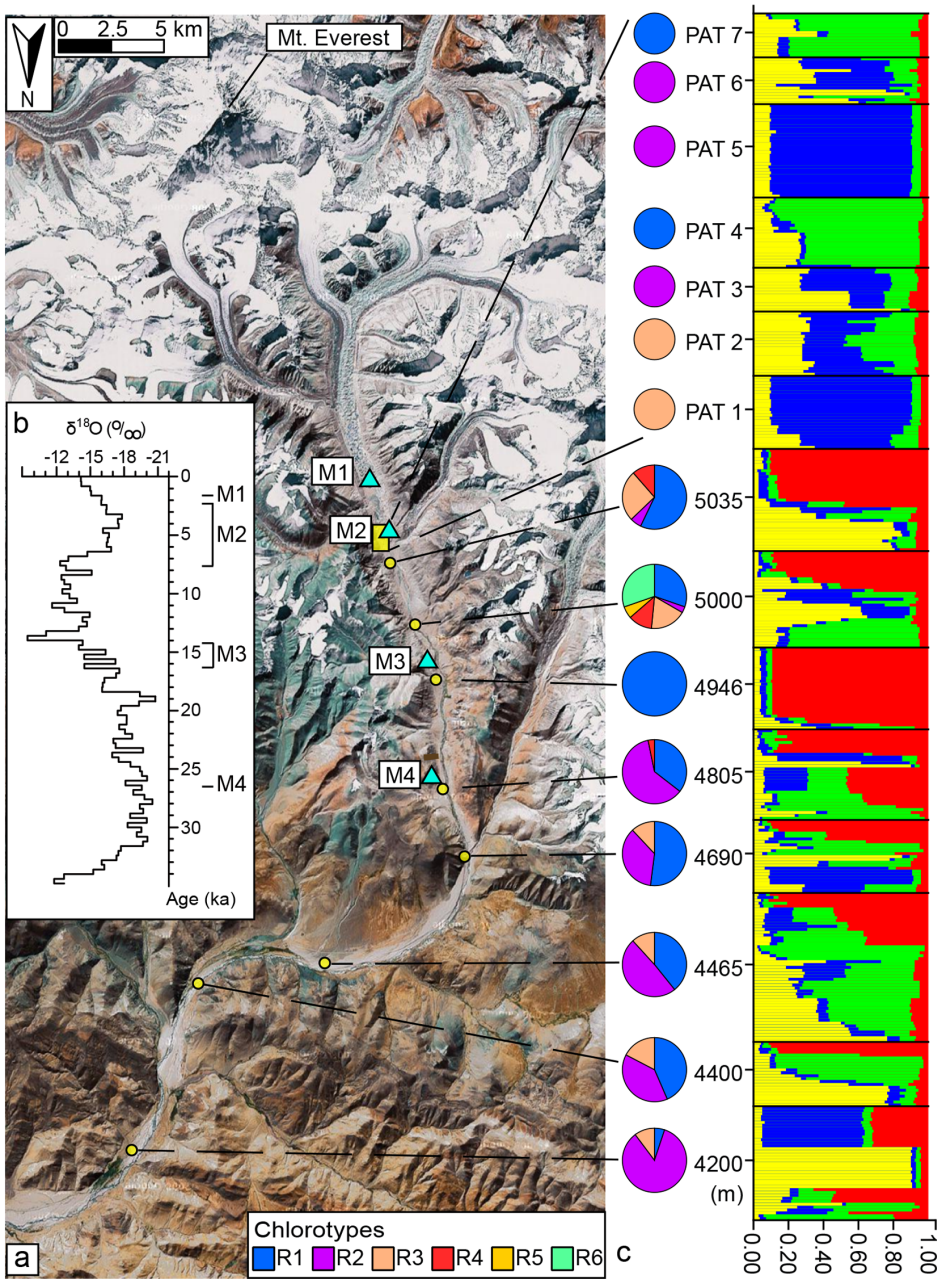
Sampling locations of *Hippophae tibetana* and the genetic composition of each population. (*a*) Map showing the sampling locations along the Rongbuk Valley, north of Mt. Qomolangma. Populations are represented by yellow circles and the patches are in the yellow rectangular area. Blue triangles represent the end-moraine of each glaciation [47], [52], [54] (M1, Little Ice Age moraine; M2, Rongbude moraine; M3 Rongbuk moraine; M4, Jilong moraine). (*b*) Temperature change revealed in the Guliya ice-core *δ* ^18^O record over the past 35 ka [38]. OSL ages of the moraines [47] are indicated along the time axis. (*c*) Elevation of each population, chlorotype composition, and proportion shown as pie charts (left column) and the population assignment test results with the software STRUCTURE (right column).

The modern Equilibrium Line Altitude (ELA) of these glaciers is above 6000 m asl [51]. In the past, their extents shifted in response to climate change, leaving terminal moraines of different ages in the valley [47], [52], [53], [54], [55] (Fig. 1a). The Far East Rongbuk ice-core only records climate changes for the last 200 years, but these can be matched with the longer Guliya ice-core records [38], [45]. We can thus infer past temperature change over a long time span in the Rongbuk Valley from the Guliya ice-core. An increase (or decrease) of 1‰ in mean annual *δ* ^l8^O in the Guliya ice-core corresponds to an increase (or decrease) of ∼1.5°C in mean annual air temperature [56] as shown in Fig. 1b. There are also detailed studies on the glacial moraines of different ages. From 25 ka B.P. until now, there have been four main glaciations in the valley, and their terminal moraines are the Jilong moraine (∼4800 m), Rongbuk moraine (∼5000 m), Rongbude moraine (∼5100 m), and the Little Ice Age moraine (∼5150 m) from the oldest to the youngest (M4, M3, M2, and M1 in Fig. 1a, respectively) [47], [52], [53], [54]. The ages of the moraines have been estimated using optically stimulated luminescence (OSL) and terrestrial cosmogenic nuclide (TCN) dating by Owen *et al*. (2009) [47] and the OSL dates are indicated along the time axis in Fig. 1b.

Main vegetation in study area is alpine steppe vegetation with small shrubs in the bottom of the valley at an altitude between 4400 and 5000 m asl, dominated by *Artemisia wellbyi*, *Stipa purpurea*, *Orinus thoroldii*, *Carex montis-everestii*, *Potentilla parvifolia*. At the higher level, between 5000–5600 m, some alpine sparse and cushion vegetation occurs locally on the upper part of area, such as *Potentilla fruticosa* var. *pumila*, *Androsace tapete*, *Arenaria polytrichoides*, *Kobresia pygmaea*, *Kobresia prainii*, *Saussurea gnaphalodes* and other *Saussurea* sp. In addition, some lichen common species occurs on the leeward side of rocks and gravels, for example *Lecidea auriculata* and *Cloplaca elegans* etc.

### Climate Conditions

In terms of climate conditions, the Himalaya and the QTP are located in the inter-tropical convergence zone. Mt. Qomolangma is influenced by the mid-latitude westerlies and the south Asian monsoon and the climate in the Rongbuk valley is semi-arid and cold [57], [58]. According to data obtained from 2008 to 2011, annual mean air temperature at Qomolangma Station (4300 m alt.) is 4.3°C and average annual precipitation is 213.4 mm [58]. The rainy season is from June to August in summer, when moisture comes with the monsoon from the Indian Ocean and the Bay of Bengal. Although it is cold in the valley, air temperature has increased in the last four decades at a rate of 0.302°C per decade (from 1971 to 2004), reflecting a high sensitivity to recent global warming [46]. This warming trend has contributed to the retreat of glaciers in recent decades [55]. In this valley, mean air temperature drops 0.7–0.8°C with a 100 m increase in altitude as a result of changes in atmospheric pressure and surface conditions [48].

### Sample Collection and Field Investigation

In the Rongbuk valley, *H. tibetana* extends from ∼4200 m to ∼5200 m asl along the banks of the river and is one of dominant species in the valley. There are some large colonies (area>1000 m^2^) between 4200∼5035 m asl, but only a few small colonies of 20–250 m^2^ areas are scattered above 5035 m (>5035 m but <5100 m asl). We are not sure that all the individuals of *H. tibetana* in this valley consist of one or some populations, so when we collected samples, we treated large colonies at different altitudes as different populations (POP) and a small colony above 5035 m as a patch (PAT). Leaves of *H. tibetana* were collected in the summers of 2007−2010. Population samples (POP) were collected along the elevational gradient from 4200 m to 5035 m every 200 m at most, and individuals are at least 10 m apart (Fig. 1a). We also surveyed the area from 5035 m to 5200 m in a ∼150-metre-wide belt transect, and collected leaf samples from all the patches of the shrub that we found (Table 2; Fig. 1a). In the patches, the spacing of individuals is not always over 10 m because of the small area of some patches. Leaves were dried in silica gel. In total, we analyzed 414 individuals from 8 populations and 7 patches, and their GPS data are shown in Table 1. Field measurements were conducted in January 2011 to determine the colonization times of the patches. Areas, crown diameters, and colonization times of the patches were estimated. The five biggest individuals in each patch and the highest large colony (POP 8 at 5035 m) were measured. The basal stems of the biggest individual in each patch and POP8 at 5035 m were cut (∼5 cm length) and conserved in plastic bags. Transverse section toward root was smoothed in the laboratory and annual rings were identified by using a dissecting microscope.

**Table 2:** Area, crown diameter, and annual ring of 5 patches and the highest population.

Patch No.	Altitude (m)	Area (m^2^)	Crown diameter (cm)^a^	Annual rings^b^
POP 8	5035	>5000	56.6±2.70	40
PAT 4	5047	∼250	26.6±3.21	37
PAT 3	5046	∼50	17.0±2.34	30
PAT 2	5047	∼30	14.4±6.95	15
PAT 7	5058	∼30	8.8±1.30	21
PAT 5	5066	∼20	8.4±1.14	15

a the mean value ± S.D. of crown diameters of five individuals with the biggest crow; ^b^the annual rings of the individual with the largest basal stem.

### Genetic Analysis

Total genomic DNA was extracted using the modified CTAB method [43]. Both the chloroplast trnT-trnF sequences and nuclear microsatellites are used in the present study.

**Figure 2:**
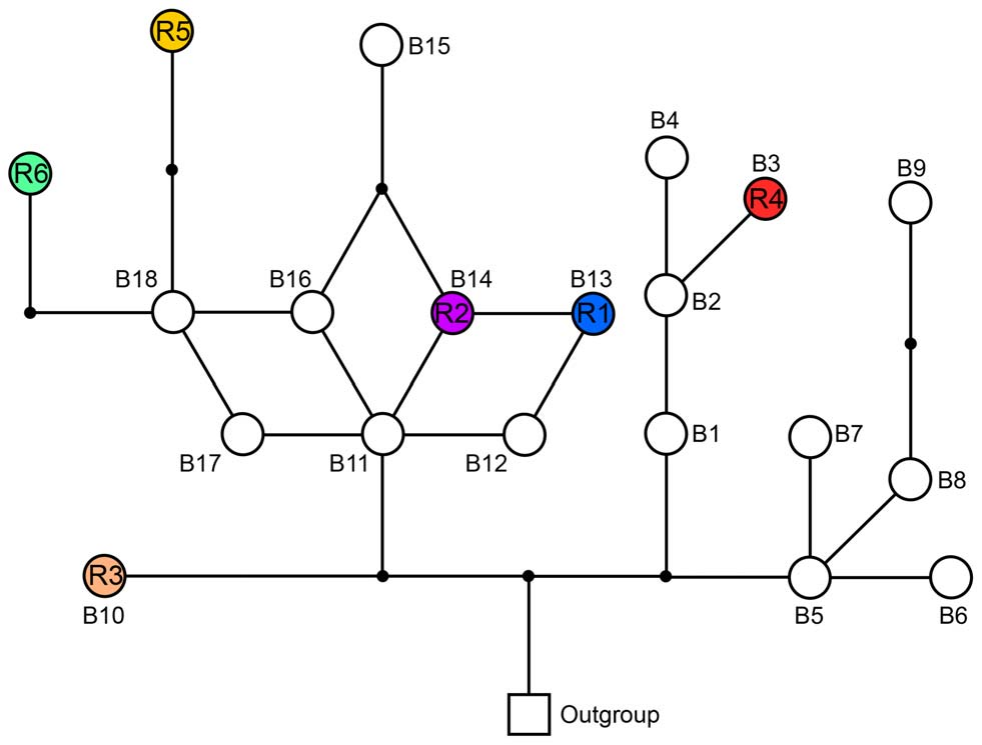
Chlorotype network based on the *trn*T-*trn*F sequences of *H. tibetana*. Circles with letters inside indicate chlorotypes found in the Rongbuk Valley. The other circles represent the remaining B-lineage chlorotypes found in the western part of the species’ geographical range and the outgroup consists of the A-lineage chlorotypes that occupy the central part of the range [43]. Genbank accession numbers of these haptotypes were listed in Table S2 in File S1.

Based on the sequences from our previous phylogeographic study [43], we selected partial *trn*T-*trn*F sequences of chloroplast DNA to identify chlorotypes. Amplification and sequencing were carried out using primers INP1 5′ TAGATCGTTCAAGTATTCAAAATA 3′ and INP2 5′ CAGGTCGTCATTAATCATTTTCAGA 3′, following the procedures of Wang *et al*. (2010). The DNA sequences of *H. tibetana* individuals were aligned using the program CLUSTAL X with subsequent manual adjustments, and assigned to different haplotypes using DnaSP 5.10 [59]. Our previous study has shown that all the *trn*T-*trn*F haplotypes of populations in the Mt. Qomolangma area and adjoining region belong to the B-lineage [43] To evaluate the evolutionary relationships among chlorotypes, the chlorotypes obtained in the present study and all the B-lineage chlorotypes from Wang *et al*. (2010) were used to construct a network by the program NETWORK version 4.5.1.0 (http://www.fluxus-engineering.com) which uses the median joining approach to combine a minimum spanning tree within a single network and then, by the criterion of parsimony, median vectors are added to the network (Fig. 2). The indices of unbiased genetic diversity (*D*) and nucleotide diversity (*π*) [60] were calculated for each population using the program ARLEQUIN version 3.1 [61]. Using part of the *trn*T-*trn*F sequences that we had obtained for all individuals, pairwise mismatch distributions were carried out with ARLEQUIN version 3.1 for populations containing different chlorotypes to infer their demographic history. The sum of the squared differences (SSD) was used as a statistical test to accept or reject the hypothesis of sudden population expansion. The raggedness index and its significance were calculated to quantify the smoothness of the observed mismatch distribution [62].

Microsatellite loci were developed using 5′-anchored PCR [63], [64] and five microsatellite loci exhibiting polymorphism among individuals from the Rongbuk Valley (Table S1 in File S1) were amplified following the procedures of Song *et al*. (2003) to analyze the genetic variation [65]. All the primers had reliable scoring and the interpretation of electropherograms was performed by the same person in the same laboratory for all samples. For each population, the intra-population genetic diversity was evaluated (Table 2). Fixation index (*F*_IS_) and allelic richness (A, mean number of alleles per locus based on the minimal sample size) were calculated with FSTAT version 2.9.3.2 [66] using five microsatellite loci, and H_E_ and H_O_ were calculated with GENETIX version 4.03 (www.genetix.univ-montp2.fr). For tests of deviation from the Hardy-Weinberg equilibrium and genotypic disequilibrium, the *p*-values obtained (with a 0.05 significance threshold) were adjusted in FSTAT version 2.9.3.2 by applying sequential Bonferroni corrections to avoid false-positives [66]. Pairwise *F*-statistics (*F*_ST_) among populations and patches were calculated using five microsatellite loci with GENETIX version 4.03, the significance of which was tested by comparison of the 95 and 99% confidence intervals derived from 1000 bootstrap permutations individuals across sites. To illustrate the shortest dispersal route between populations, we connected populations on a non-metric multidimensional scaling (NMDS) of *F*_ST_ using a minimum-spanning tree (MST) [67]. The bottleneck test was performed using BOTTLENECK version 1.2.02 [68] with five microsatellite loci. We employed the Wilcoxon sign-rank test because of the small number of loci [69], [70]. The estimates are based on 1000 simulations performed under both the strict one-step stepwise mutation model (SMM) and the two-phase model (TPM: 70% single-step mutation and 30% multi-step mutation) assumptions. A Bayesian clustering approach was used to infer population structure with STRUCTURE version 2.2 [71]. We used the admixture model and correlated allele models without any prior information. Twenty independent runs with a burn-in of 10,000 and 100,000 Markov chain Monte Carlo simulations for each value of *K* (from 1 to 15) were used to evaluate the genomic composition of the populations. We then used the Δ*K* statistics to evaluate the change in likelihood [72].

## Results

### Size of Patch and Annual Rings of the Biggest Individual

The sizes of the patches above 5035 m asl and the annual rings of the biggest individual in each patch are shown in Table 2. Five out of seven patches were found in the field investigation of 2011 according to the previous GPS data, but PAT 1 and PAT 6 could not be re-found. The topography of the sample region is rugged and some patches are small, thus we guess that the inaccurate GPS data of some patches and the harsh weather in January 2011 led us not to re-find these two patches.

The basal stems of all the cut individuals have clear annual rings when viewed under a dissecting microscope. The age (annual ring) of the biggest individual of the large colony (POP8) at 5035 m is 40 years and that of the largest patch at 5047 m is 37 years. The patch with the smallest size and crown diameter, PAT 5, grows at the highest altitude, 5066 m, and has the youngest age (15 years). The crown diameter of patch is related negatively to its altitude (R^2^ = 0.69, *F* = 8.83, *p* = 0.041), and the same relationship exists between the annual ring of the biggest individual and its altitude (R^2^ = 0.56, *F* = 5.14, *p* = 0.086).

### Chlorotypes and Distribution

Chloroplast DNA *trn*T-*trn*F sequences of 414 individuals from 8 populations (large colonies) and 7 patches (small colonies over 5035 m asl) at different elevations in the Rongbuk Valley were sequenced in this study and six chlorotypes were identified (Fig. 1c and Table 1). Genebank accession numbers of the chlorotypes are R1 - GU561447, R2 - GU561448, R3 - GU561444, R4 - GU561441, R5 - JF268789, and R6 - JF268790. Among these six chlorotypes, R5 and R6 were first found in this study and the others (R1–R4) were reported in our previous study [43]. Comparing with all the chlorotypes found in *H.tibetana* [43], four of these six haplotypes, R1, R4, R5 and R6, are endemic to this valley, i.e. private haplotypes. The chlorotype compositions of the populations at different elevations are different (Fig. 1c). All the populations below 4700 m contain the same chlorotype component: three chlorotypes (R1, R2, R3), while the population at 5000 m has all six chlorotypes (Fig. 1c); the patches over 5035 m also have three chlorotypes (R1, R2, R3), but each patch consists of only one (Fig. 1c). Unbiased genetic diversity (*D*) within the populations in the Rongbuk Valley ranges from 0 to 0.7879, and nucleotide diversity (*π*) from 0 to 0.00343 (Table 1). A network of the six chlorotypes and other chlorotypes occurred in the west part of the species’ geographical range (the B-lineage chlorotype, sequences from Wang et al. 2010) shows that chlorotypes found in the Rongbuk Valley are scattered within the network (Fig. 2).

### Genetic Structure

**Figure 3:**
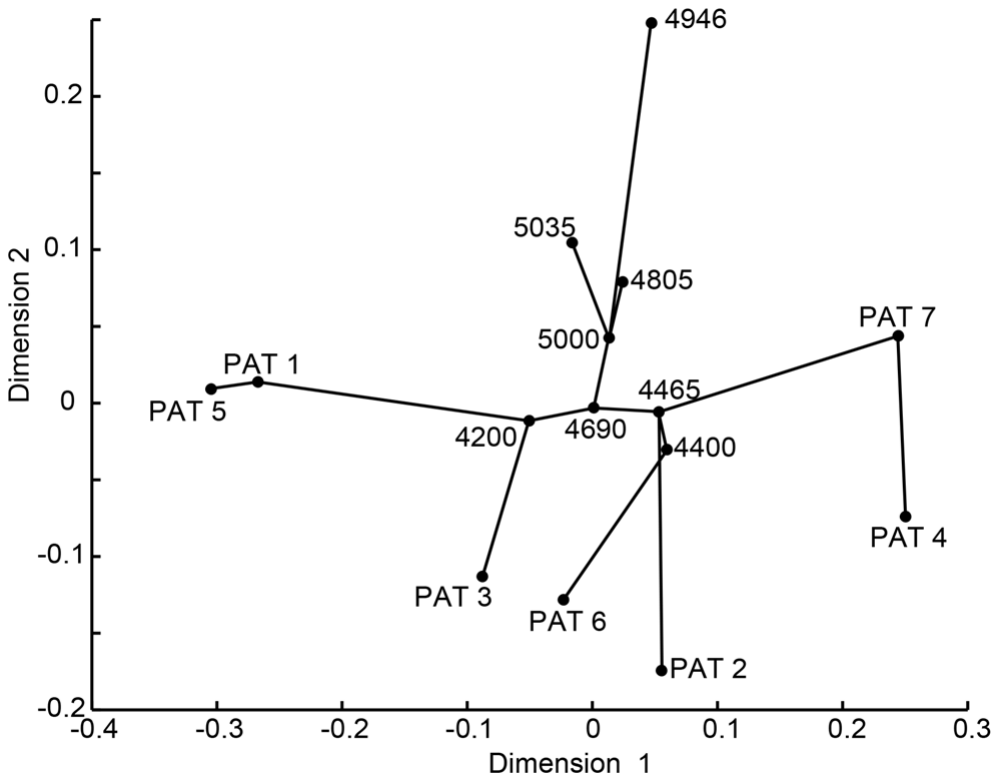
Non-metric multidimensional scaling of pairwise *F*_ST_ and a minimum-spanning tree linking populations and patches. Populations are indicated by their altitudes (m).

Five microsatellite loci exhibiting polymorphism among individuals from the Rongbuk Valley were obtained. Their primer sequences are listed in Table S1 in File S1. Bayesian clustering of the information from the five microsatellite loci demonstrates that the model with *K* = 4 explains the data satisfactorily based on the Δ*K* statistics. Each patch contains genetically similar individuals revealed by the STRUCTURE analysis, whereas populations usually have more complex components except for the population at 4946 m (Fig. 1c). All the patches and the 4946 m-population also exhibit large negative *F*_IS_ values and a significant deviation from the Hardy-Weinberg equilibrium (Table 1). Pairwise *F*_ST_ shows little differentiation between populations except for the 4946 m-population (Table 3). The NMDS projection accounts for 88.5% (stress = 0.115) of the variance in the *F*_ST_ matrix (Table 3, Fig. 3). The 4690 m-population and the 5000 m-population are at the centre of the MST; populations higher than 4690 m are all linked to the 5000 m-population while others are linked to the 4690 m-population. All the patches are connected to populations below 4690 m in the MST (Fig. 3).

**Table 3:** Genetic differences (*F*st) among populations and patches using data from five microsatellite loci (Table S1).

FST	POP2	POP3	POP4	POP5	POP6	POP7	POP8	PAT1	PAT2	PAT3	PAT4	PAT5	PAT6	PAT7
POP1	0.0603^ns^	0.0928**	0.0303^ns^	0.0884^ns^	0.1950**	0.0488^ns^	0.0631^ns^	0.2147**	0.1671**	0.0403^ns^	0.2473**	0.2336**	0.0552^ns^	0.2564**
POP2		0.0130^ns^	0.0158^ns^	0.0632^ns^	0.2196**	0.0377^ns^	0.0860^ns^	0.2948**	0.0863**	0.1308**	0.1425**	0.3274**	0.0436^ns^	0.1157**
POP3			0.0119^ns^	0.0525**	0.1609**	0.0200^ns^	0.0834**	0.2351**	0.0715**	0.1225**	0.1542**	0.2664**	0.0897**	0.0751^ns^
POP4				0.0274^ns^	0.1638**	−0.0006^ns^	0.0548^ns^	0.1820**	0.0876**	0.0718^ns^	0.1757**	0.2096**	0.0512^ns^	0.1592**
POP5					0.1503**	0.0068^ns^	0.0345^ns^	0.2583**	0.1864**	0.1882**	0.1994**	0.2866**	0.1632**	0.1923**
POP6						0.1244**	0.1254**	0.4405**	0.3579**	0.2944**	0.3667**	0.4813**	0.3480**	0.3050**
POP7							0.0253^ns^	0.2024**	0.1357**	0.1138**	0.1767**	0.2330**	0.1144**	0.1450**
POP8								0.2835**	0.2057**	0.1794**	0.2997**	0.3171**	0.1759**	0.2464**
PAT1									0.3075**	0.2777**	0.4712**	0.0054^ns^	0.2863**	0.5185**
PAT2										0.2148**	0.3252**	0.3430**	0.0785^ns^	0.2545**
PAT3											0.3123**	0.2995**	0.0710^ns^	0.3420**
PAT4												0.5007**	0.2666**	0.1225**
PAT5													0.3066**	0.5612**
PAT6														0.2920**

The *F*-statistics (*F*_ST_) values were calculated with GENETIX version 4.03, the significance of which was tested by comparison of the 95 and 99% confidence intervals derived from 1,000 bootstrap permutations individuals across sites.

ns *p*>0.05,

*p≤0.05,

**p<0.001.

### Population Expansion and Population Bottleneck

Results of the Wilcoxon sign-rank test and the mismatch distribution analysis are given in Table 4. The Wilcoxon test values under both the strict one-step stepwise mutation model (SMM) and the two-phase model (TMP) are significant in POP 1, 2, 5, 7, and 8, indicating that these five populations have been through recent bottlenecks. Furthermore, when performing mismatch analysis, the SSD *p*-value and raggedness index for POP 1, 2, 4, 5, and 7 are not significant at the 95% significance level, suggesting that these five populations have experienced demographic expansion (Table 4).

**Table 4:** Bottleneck tests and mismatch distribution analysis.

Population No.	Altitude (m)	Wilcoxon Test		Mismatch distribution analysis	
		SMM	TPM	SSD *p*-value	Raggedness index
POP 1	4200	0.0469*	0.0312*	ns	0.273^ns^
POP 2	4400	0.0312*	0.0156*	ns	0.164^ns^
POP 3	4465	0.3125^ns^	0.3125^ns^	0.028	0.195*
POP 4	4690	0.5000^ns^	0.3125^ns^	ns	0.148^ns^
POP 5	4805	0.0469*	0.0312*	ns	0.218^ns^
POP 6	4946	0.0625^ns^	0.0625^ns^	–	–
POP 7	5000	0.0312*	0.0312*	ns	0.067^ns^
POP 8	5035	0.0312*	0.0312*	ns	0.476*
PAT 1	>5035	0.2500^ns^	0.2500^ns^	–	–
PAT 2	5047	0.1250^ns^	0.1250^ns^	–	–
PAT 3	5046	0.1250^ns^	0.1875^ns^	–	–
PAT 4	5047	0.0625^ns^	0.0625^ns^	–	–
PAT 5	5066	0.2500^ns^	0.2500^ns^	–	–
PAT 6	>5035	0.1250^ns^	0.1250^ns^	–	–
PAT 7	5058	0.1250^ns^	0.1250^ns^	–	–

(^ns^ *p*>0.05, *p≤0.05, **p<0.001).

## Discussion

### The Microrefugia of *H. tibetana* in the Rongbuk Valley

In previous study, we inferred that the Rongbuk Valley may have been a LGM microrefugium for *H.tibetana* based on private chlorotypes [43]. In the present study, we analysed 414 individuals from this valley using chloroplast DNA *trn*T-*trn*F sequences and five nuclear microsatellite loci. More private chlorotypes were found and their distribution in the valley was revealed. By comparing chlorotypes here to those of *H. tibetana* elsewhere, including the two known nearest populations in the Mt. Qomolangma area (Dingri population and Nielamu population, see reference 43), we found that four (R1, R4, R5, R6) of six haplotypes found in the Rongbuk Valley are endemic to this valley, i.e. private haplotypes. In addition, these six chlorotypes are scattered in the chlorotype network (Fig. 2), and their divergence time could date back to 1.52±0.814 million years ago [43]. Considering the Rongbuk Valley is so high and had dramatically influenced by glaciations during the LGM [47], [49], the fact that so many private chlorotypes occur here is surprising. These private haplotypes only have three potential origins: origination from common haplotypes (R2 and R3) after the LGM, coming from other areas after the LGM, and survival through the LGM in the valley. As Opgenoorth et al. discussed in the previous study on *Juniperus tibetica* complex [22], these private chlorotypes are very unlikely to have originated from common haplotypes after the LGM according to the substitution rate of cpDNA *trn*T*-trn*F regions (from the fastest, 8.24×10^–9^ substitutions per site per year, to the lowest, 4.87×10^–10^ ) [73]. Also, they are very unlikely to have come recently from other areas because in these other areas, including regions very near this site, the above private chlorotypes have not found according to our previous study [43]. Thus, the third origin, survival in the valley through the LGM is the only acceptable hypothesis. These results provide strong evidence that the Rongbuk valley was a refuge for *H.tibetana* during the LGM and possibly earlier glaciations. According to the definition of microrefugium [9], it is a microrefugium for *H.tibetana* because the Rongbuk valley is far away from its main range and a very small part of its whole range.

### Shifts of *H. tibetana* in the Last 25,000 Years

As the 5000 m-population has all six chlorotypes and the highest genetic diversity (*D* and *π* in Table 1), including two endemic types (R5 and R6) in this population, it seems that in the valley, the microrefugium for *H. tibetana* in the past was located in the area at 5000 m. However, the integration of geological [47], [52], [54], glaciological [38], [45], [55], meteorological [46], [48], and genetic information reveals that the survival process may have been complex. From 25 ka B.P. until today, there have been four major periods of glacier advance that formed four terminal moraines in the Rongbuk valley (M1-M4, Fig. 1a) [52]. A terminal- or end-moraine, forms at the end of the glacier tongue and marks the maximum advance of the glacier. During the LGM, the valley area over 4800 m was covered by the glacier tongue, which consists of ice and gravel and would prevent *H. tibetana* from colonizing. The valley, except riversides and riverbed, is very arid today because of its special topography (46) and *H. tibetana* is not able to survive on the xeric side slopes in the valley. During the glacial stages, the QTP became more arid [74], [75], and the valley would be more arid. Thus, the xeric side slopes in the valley were not suitable habitats for *H. tibetana.* In fact, all populations of *H. tibetana* found in our field investigation and reported by other collectors did not occur over glacier tongues. These results support that the upper limit of *H. tibetana* in the valley must have been below the glacier tongue.

According to data from the Guliya ice-core (Fig. 1b), temperature during the LGM was ∼9°C lower than present [38], [56], and the glacier tongue dropped to ∼4800 m [47], [54] (M4 in Fig. 1a, b), so the upper limit of *H. tibetana* must then have been below 4800 m. According to the altitudinal difference between the recent glacier tongue and the recent upper limit of *H. tibetana* in this valley (∼200 m), we infer that the upper limit of *H. tibetana* during the LGM might have been about 4600 m. It means that the microrefugium for *H. tibetana* during the LGM was located below 4800 m, and all six chlorotypes of *H. tibetana* should occur in the area below 4800 m. But now, though we checked all the populations below 4800 m, three private chlorotypes (R4, R5, and R6) could not be found in this area. This fact indicates that after the LGM, populations of *H. tibetana* in the LGM microrefugium, i.e. climate relict according to Hampe & Jump (2011) [24], have already undergone dramatic contraction or extinction. From the LGM to the Younger Dryas (YD, a cold episode around 12 ka ago), temperature fluctuated several times [38]. Annual average temperature at ∼14.2 ka BP may have been up to 7°C higher than present, but it decreased dramatically by ∼10°C around 12 ka BP, and suddenly increased by 4.5°C about 11 ka BP according to data from the Guliya ice-core (Fig. 1b). The glacier formed the conspicuous Rongbuk end-moraine complex (5000 m, M3 in Fig. 1a). After the YD, there was a ∼4 ka warm period (∼11 to 7 ka ago, Fig. 1b) and temperature in this period was ∼3°C higher than present (Fig. 1b). Although these rapid and dramatic climate fluctuations were very likely to drive *H. tibetana* to shift up and down in the valley, the history of this species from the LGM to the YD in this valley is difficult to be proved. But at least during the warm period after the YD (∼11 to 7 ka ago, Fig. 1b), *H. tibetana* had migrated upward to 5000 m. The reasons are as follows: 1) only the 5000 m-population now has the highest genetic divergence with all six chlorotypes, 2) two of the six chlorotypes, R5 and R6, are endemic to this 5000 m-population, strongly indicating that not less than these two endemic chlorotypes had colonized at this altitude during this period, and 3) these two endemic chlorotypes are unlikely to originate here from other chlorotypes in such a short period (Fig. 2). It means that from the LGM to the warm time after the YD, the upper limit of *H. tibetana* shifted at least 200 m upward, and most probably over 400 m (from ∼4600 m to 5000 m asl or higher). Meanwhile, the lower limit of this species here might upward shift to 4800 m or higher in this warm period, and at least populations in the LGM microrefugium have undergone extreme contraction, which resulted in the loss of three chlorotypes (R4, R5, R6) in the LGM microrefugium. These results demonstrate that the area about 5000 m plays an interglacial refuge and that some area below 4800 m was merely a glacial refuge.

When populations shift up and down repeatedly, populations will undergo genetic bottlenecks and expansions [76]. These genetic events have been observed in most populations based on microsatellite and chlorotype data, respectively (Table 4). Analysis of microsatellites provides us with more information. Non-metric multidimensional scaling of pairwise *F*-statistics (Table 3) and a minimum-spanning tree show that the 4690 m-population and the 5000 m-population are at the centre of the net (Fig. 3), and that populations higher than 4690 m are all linked to the 5000 m-population while others are linked to the 4690 m-population. These results imply that three present populations below 4690 m are the result of the expansion of populations at higher altitude, and that the lower limit of *H. tibetana* moved upward to at least 4690 m when climate became warmer after the LGM, especially after the YD.

Information from both chlorotypes and microsatellites strongly demonstrate that the genetic structure of populations of *H. tibetana* in the LGM microrefugium has been changed greatly after the LGM. If we infer glacial microrefugia only based on genetic information, we are likely to be misled, because present populations in glacial microrefugia may not be true climate relicts of glacial stages. Similar patterns have been found at a broader scale. The study on chloroplast DNA variation in 22 widespread European trees and shrubs sampled in the same forests has shown that the genetically most diverse populations were not located in glacial refugia in southern Europe but at intermediate latitudes [77]. The authors proposed that it was a likely consequence of the admixture of divergent lineages colonizing the continent from separate refugia. We suspect that present populations in glacial refugia in southern Europe have not been true glacial relics.

### Recent Shifts of *H. tibetana*

In the last 100 years, altitudinal ranges of many plants have shifted upward, presumably in response to global temperature increases [29], [32], [33], [37], [78]. The Mt. Qomolangma area is one of the most vulnerable areas to global change [79], [80], [81], and in the last four decades (from 1971 to 2004), annual mean temperature has increased here at a rate of 0.302°C per decade and from 1976 to 2006, the ends of Central Rongbu glacier has retreated about 500 m in length [55], while precipitation has stayed at a constant level [46]. In our field investigation, we only found seven scattered patches in the region over 5035 m (Table 1). These patches are mostly located on the Rongbude terminal moraine (M2, Fig. 1a) and have not been covered by landslides. No dead patches could be found in this region and few or no dead individuals could be found in these patches. These results show that these patches are not the relict of an old large colony but new small colonies, and that the annual ring of the largest individual of a patch can indicate the successful colonization age of this patch. These patches have a colonization age of 15∼37 years and show a significant negative relationship between colonization time and altitude, i.e. the higher the altitude of the patch, the younger the patch (Table 2), demonstrating that these patches must have colonized here in the last 40 years and that these patches shifted step by step from lower to higher sites. The highest large colony (POP 8) is located at 5035 m, and the highest patch (PAT 5) at 5066 m. In fact, the annual ring of PAT 5 at 5066 m is 15 years, indicating that *H. tibetana* had arrived at 5066 m 15 years ago and that the shift of *H. tibetana* from 5035 m to 5066 m only spent about 25 years. This rate is slower than the mean rate of shift of alpine plants above tree-line in the European Alps and of forest plants in western Europe (27.8 m per decade; 29.4 m per decade) [33], [82]. The shift of *H. tibetana* in the valley during the past 40 years is also smaller than the inferred value (∼100 m) based on an average decrease of 0.7∼0.8°C per 100 m increase in altitude and an average increase of 0.302°C per decade in the Qomolangma area [46], [48]. Lenoir *et al.* (2008) showed that species with different ecological properties displayed different rates of shift and that small grasses moved faster than large woody plants [33]. We suspect that the high altitude (over 5000 m) of our study area may influence the upward rate of shift of plants. Grabherr *et al*. (1994) found that mountain plants that grew above 3000 m in the central part of the European Alps have slower rates of shift (<4 m per decade) than those that grow below 3000 m [29]. Besides climate warming and glacier shrinkage, precipitation and other ecological factors have not obviously changed during the past 40 years in the Rongbuk Valley, and human activity was very infrequent in this valley 20 years ago [52], [46]. Thus, global warming is likely to be the main reason for the recent upward shift of *H. tibetana*.

This study also highlights the colonization pattern of primary habitats. Each patch contains only one chlorotype and consists of highly genetically similar individuals inferred from the microsatellite data (Fig. 1c), indicating that all the patches expanded mainly by clonal reproduction, which then resulted in a deviation from the Hardy-Weinberg equilibrium and high *F*_ST_ values (Table 1). Although the 4946 m-population area is much larger than the patches, it is on a landslide and almost entirely consists of small and genetically identical individuals (Fig. 1c, Table 1), implying it is also a new clonal population after the landslide formed.

### Factors Determining the Survival of *H. tibetana* in the Rongbuk Valley

Plant survival depends on a favourable local environment [9], [15], and extreme conditions, stable climates, and distinct differences from the surrounding matrix are the basis for microrefgia [19]. According to detailed topoclimate, Ashcroft et al. (2012) deduced that a mountain area may have not only warm refugia but cool refugia for striking differences in climate among different locations [19]. In the Rongbuk Valley, mountains play an important role in the ability of *H. tibetana* to withstand climate changes. Here, the elevational range of the valley is nearly 1500 m (from 3700 m to 5200 m asl) and mean temperature drops 0.7–0.8°C with each 100 m increase in altitude in this area [48], implying that there is nearly a ∼11°C temperature gradient, which not only provides *H. tibetana* with a wide range within which to shift its occurrence in response to climate change, but also provides a series of fine-scale ecological niches with different microclimates that can buffer against temperature fluctuations [15], [83], [84]. In fact, with the retreat of glaciers in warmer periods, the area over 5200 m in the valley is likely to become a suitable habitat for *H. tibetana*.

In addition, glaciers are critical for *H. tibetana*. Historically when it became cold on the QTP, widespread aridity also occurred [74], and the special topography of the Mt. Qomolangma area caused the Rongbuk Valley to receive lower rainfall and experience more droughts. Glacial meltwater have thus provided almost all the moisture for *H. tibetana*. Our field investigation also confirms the importance of glaciers. In the semi-arid and arid areas in the QTP, all the eleven populations of *H. tibetana* were located in the valley below large glaciers except for the Ritu population [43], which was once near glacier [85] but the population is now shrinking as the local glacier disappears. Besides *H. tibetana*, there are many other plants which mainly depend on glacial meltwater for their moisture sources in the Rongbuk Valley [52]. We thus think that this valley may have been a favourable microhabitat for some of these plants in both cold and/or warm stages. Furthermore, in arid areas on the QTP, water is a limiting factor for the growth of many plants [86]. Similar to the Rongbuk Valley, water supply depends largely on glaciers in many places on the Plateau [87]. In these areas, glaciers become a crucial resource for the growth of many plants not only in warm periods but also in cold glacial stages.

Taken together, we present evidence to indicate the Rongbuk Valley, Mt. Qomolangma is a microrefugium of the LGM for *H. tibetana*, and how this plant here has responded to climate changes in the last 25 ka. By combining historical and recent range shifts, we have derived a better understanding of the rates of range shift, the dynamics of populations in the microrefugium after the LGM, and the mode of colonization of *H. tibetana*, all of which are important in inferring the potential locations of microrefugia for *H. tibetana* and predicting the response of this endemic species in relation to future climate warming.

## Supporting Information

File S1 **Tables S1–S2.** Table S1. Characterization of polymorphic microsatellite loci in this study. PCR products were electrophoresed on 7.5 m urea 6% polyacrylamide gel, sized with the DNA ladder pUC19/MspI (Fermentas Life Sciences) and visualized by silver stain as in ref 36. All the primers had reliable scoring. Ta, annealing temperature; NA, number of alleles; HO, observed heterozygosity; HE, expected heterozygosity. HO and HE were calculated with Genetix version 4.03 (www.genetix.univ-montp2.fr). When POP 7 and the seven patches were not counted because they are most probably new clone populations, no significant Hardy–Weinberg disequilibrium was detected for each locus (for the seven remaining populations each or as a whole). Table S2. Genbank accession numbers of the haptotypes in Fig. 2. Haptotype B1–B18 and the haptotypes of outgroup were found in our previous work (reference 43). The outgroup consists of Haptotype A1–A6. R5 and R6 were found in the present study.
(DOCX)
