# Supplementary material for: Microrefugia and Shifts of Hippophae tibetana (Elaeagnaceae) on the North Side of Mt. Qomolangma (Mt. Everest) during the Last 25000 Years
Source: PLoS One. 2014 May 19;9(5):e97601. doi: 10.1371/journal.pone.0097601 (PMC4026410; doi:10.1371/journal.pone.0097601)
Supplement: File S1 — Tables S1–S2. Table S1. Characterization of polymorphic microsatellite loci in this study. PCR products were electrophoresed on 7.5 m urea 6% polyacrylamide gel, sized with the DNA ladder pUC19/MspI (Fermentas Life Sciences) and visualized by silver stain as in ref 36. All the primers had reliable scoring. Ta, annealing temperature; NA, number of alleles; HO, observed heterozygosity; HE, expected heterozygosity. HO and HE were calculated with Genetix version 4.03 (www.genetix.univ-montp2.fr). When POP 7 and the seven patches were not counted because they are most probably new clone populations, no significant Hardy–Weinberg disequilibrium was detected for each locus (for the seven remaining populations each or as a whole). Table S2. Genbank accession numbers of the haptotypes in Fig. 2. Haptotype B1–B18 and the haptotypes of outgroup were found in our previous work (reference 43). The outgroup consists of Haptotype A1–A6. R5 and R6 were found in the present study. (DOCX) [file pone.0097601.s001.docx]

**Table S1**. **Characterization of polymorphic microsatellite loci in this study**. PCR products were electrophoresed on 7.5 m urea 6% polyacrylamide gel, sized with the DNA ladder pUC19/MspI (Fermentas Life Sciences) and visualized by silver stain as in ref 36. All the primers had reliable scoring. *T*_a_, annealing temperature; *N*_A_, number of alleles; *H*_O_, observed heterozygosity; *H*_E_, expected heterozygosity. *H*_O_ and *H*_E_ were calculated with Genetix version 4.03 (www.genetix.univ-montp2.fr). When POP 7 and the seven patches were not counted because they are most probably new clone populations, no significant Hardy–Weinberg disequilibrium was detected for each locus (for the seven remaining populations each or as a whole).

| Locus | Genebank Accession No. | Primer sequence (5´-3´) | Repeat motif | *T*_a_ (℃) | Size range (bp) | *N*_A_ | *H*_E_ | *H*_O_ |
| --- | --- | --- | --- | --- | --- | --- | --- | --- |
| HS1 | JF268791 | AACCACAGCAAAACAAAAAAC  TAAAAATACACCTCCAACTCA | (TGA)_8_ | 47 | 220-234 | 3 | 0.241 | 0.337 |
| HS2 | EU429318 | CCATCCACATTCCTCTTCAA  GTCATTACCCACCTTCACAT | (GAATGT)_3_ | 45 | 120-130 | 2 | 0.109 | 0.130 |
| HS3 | EU429317 | CCCCCTTCTTTTTCAGATAGT  GAGAGTTGCATTTTTGCCCTTT | (A)_10_ | 52 | 120-129 | 3 | 0.389 | 0.613 |
| HS4 | EU429310 | CAATTGTTCAATACTAAATG  ATCCTAATCAAAAGAAATC | (A)_6_(CAAACA)_3_ | 42 | 110-123 | 3 | 0.516 | 0.623 |
| HS5 | EU429312 | TGCCAGAAGATTAGACTTTTAC  GGAGCAGCTTATACCCATTAC | (A)_8_(GAA)_4_ | 55 | 74-82 | 2 | 0.229 | 0.181 |

**Table S2** **Genbank accession numbers of the haptotypes in Fig. 2**. Haptotype B1 - B18 and the haptotypes of outgroup were found in our previous work (reference 43). The outgroup consists of Haptotype A1 - A6. R5 and R6 were found in the present study.

| Haptotype | Genbank Accession No. | Distribution |
| --- | --- | --- |
| A1 | GU561460 | Mozhugongka |
| A2 | GU561458 | Dingqing |
| A3 | GU561459 | Dingqing |
| A4 | GU561461 | Chayu |
| A5 | GU561462 | Baqing |
| A6 | GU561463 | Baqing |
| B1 | GU561443 | Pulan |
| B2 | GU561442 | Pulan, Cuoqin |
| B3 (R4) | GU561441 | Dingri |
| B4 | GU561440 | Dangxiong |
| B5 | GU561457 | Langkazi |
| B6 | GU561454 | Dangxiong |
| B7 | GU561456 | Dangxiong |
| B8 | GU561455 | Dangxiong |
| B9 | GU561453 | Dangxiong |
| B10 (R3) | GU561444 | Dingri, Nielamu |
| B11 | GU561446 | Dingri, Jilong |
| B12 | GU561445 | Dingri, Jilong, Nielamu |
| B13 (R1) | GU561447 | Dingri |
| B14 (R2) | GU561448 | Dingri, Jilong |
| B15 | GU561449 | Dangxiong |
| B16 | GU561452 | Nielamu |
| B17 | GU561450 | Pulan, Ritu |
| B18 | GU561451 | Pulan |
| R5 | JF268789 | Dingri |
| R6 | JF268790 | Dingri |
